# A stakeholder-driven approach to designing a peer recovery coach role for implementation in community-oriented primary care teams in South Africa

**DOI:** 10.21203/rs.3.rs-4566640/v1

**Published:** 2024-07-18

**Authors:** Bronwyn Myers, Kristen S. Regenauer, Kim Johnson, Imani Brown, Alexandra Rose, Nonceba Ciya, Sibabalwe Ndamase, Yuche Jacobs, Morgan Anvari, Abigail Hines, Dwayne Dean, Rithika Baskar, Jessica Magidson

**Affiliations:** Curtin enAble Institute, Curtin University; Department of Psychology, University of Maryland; Mental Health, Alcohol, Substance Use and Tobacco Research Unit, South African Medical Research Council; Department of Psychology, University of Maryland; Department of Psychology, University of Maryland; Mental Health, Alcohol, Substance Use and Tobacco Research Unit, South African Medical Research Council; Mental Health, Alcohol, Substance Use and Tobacco Research Unit, South African Medical Research Council; Mental Health, Alcohol, Substance Use and Tobacco Research Unit, South African Medical Research Council; Department of Psychology, University of Maryland; Department of Psychology, University of Maryland; Department of Psychology, University of Maryland; Department of Psychology, University of Maryland; Center for Substance Use, Addiction & Health Research, University of Maryland

**Keywords:** stigma, substance use, implementation science, task-sharing, low-and-middle income country, global mental health

## Abstract

**Introduction:**

In South Africa (SA), community-oriented primary care (COPC) teams work to re-engage out-of-care people with HIV (PWH) in treatment, many of whom have substance use (SU) concerns. SU stigma is high among these teams, limiting care engagement efforts. Integrating peer recovery coaches (PRCs) into COPC teams could shift SU stigma and improve patients’ engagement in care. The PRC role does not exist in SA and represents a workforce innovation. To enhance acceptability, feasibility, and appropriateness for the local context, we engaged multiple stakeholder groups to co-design a PRC role for COPC team integration.

**Methods:**

We used a five-step human-centered design process: (i) semi-structured interviews with healthcare worker (HCW, *n* = 25) and patient (*n* = 15) stakeholders to identify priorities for the role; (ii) development of an initial role overview; (iii) six ideation workshops with HCW (*n* = 12) and patient (*n* = 12) stakeholders to adapt this overview; (iv) refinement of the role prototype via four co-design workshops with HCW (*n* = 7) and patient (*n* = 9) stakeholders; and (v) consultation with HIV and SU service leaders to assess the acceptability and feasibility of integrating this prototype into COPC teams.

**Results:**

Although all stakeholders viewed the PRC role as acceptable, patients and HCWs identified different priorities. Patients prioritized the care experience through sharing of lived experience and confidential SU support. HCWs prioritized clarification of the PRC role, working conditions, and processes to limit any impact on the COPC team. A personal history of SU, minimum 1 year in SU recovery, and strong community knowledge were considered role prerequisites by all stakeholders. Through the iterative process, stakeholders clarified their preferences for PRC session structure, location, and content and expanded proposed components of PRC training to include therapeutic and professional work practice competencies. Service leaders endorsed the prototype after the addition of PRC integration training for COPCs and PRC mentoring to address community and COPC dynamics.

**Conclusion:**

Stakeholder engagement in an iterative HCD process has been integral to co-designing a PRC role that multiple stakeholder groups consider acceptable and that COPC teams are willing to implement. This offers a methodological framework for other teams designing SU workforce innovations.

## Background

Although South Africa (SA) has significantly expanded access to antiretroviral therapy (ART) for people with HIV (PWH), less than two-thirds of PWH in South Africa attain viral suppression (1). Intermittent or discontinued HIV treatment contributes to sub-optimal viral suppression rates (2), with only 38% of PWH continually engaging in ART during the first 12 months after initiation (3).

In response, the South African Department of Health has introduced ward- or community-oriented primary care (COPC) teams to bridge gaps between out-of-care patients and clinic-based HIV services (4, 5). COPC teams are linked to a primary care clinic and comprises an enrolled nurse outreach team leader and community health workers (CHWs) (6). These COPCs provide up to 400 households in their clinic’s geographical catchment area with HIV treatment supports, including support for out-of-care PWH to re-engage with clinic-based services and referral to health and social services for co-occurring conditions that affect HIV treatment engagement (6, 7).

One co-occurring condition that CHWs are likely to encounter when working with out-of-care PWH is substance use (SU). SU is highly prevalent in SA (8), particularly in the Western Cape province where population-level rates of SU disorders are significantly higher than the rest of the country (9). Although the prevalence of SU among PWH is unknown, approximately a third of PWH attending clinics in the Western Cape are estimated to have SU difficulties (10). As SU is associated with sub-optimal ART adherence and treatment disengagement (11, 12), the prevalence of SU difficulties may be even higher among out-of-care PWH receiving COPC.

Yet CHWs are not routinely trained to screen for SU, limiting their ability to assist out-of-care PWH struggling with this care engagement barrier (13–15). While there have been recent initiatives to train CHWs and other healthcare workers (HCW) to conduct screening and brief interventions for SU (16), high levels of SU stigma among CHWs (17, 18) may limit the potential benefits of this training for HIV care engagement.

SU stigma among CHWs and other HCWs affects access to effective HIV care (17, 19, 20). Evidence suggests that HCWs with high levels of SU stigma are less likely to provide evidence-based interventions or person-centered care (21–24). In addition, anticipated and enacted SU stigma affects PWH’s readiness for HIV care engagement (25–27). Therefore, SU stigma needs to be addressed for CHWs to effectively support people with SU to re-engage in HIV treatment.

Converging evidence suggests that SU stigma reduction interventions involving sustained social contact with people in SU recovery have the largest and most durable effects on HCW stigma (22, 28). Consequently, peer recovery coaches (PRCs) —trained individuals with lived experience of SU—are increasingly being integrated into primary healthcare teams in the US as a strategy for expanding their capacity to link patients with SU to care (29, 30). In these teams, PRCs work directly with patients to provide personalized support for SU recovery and SU treatment and health care navigation. US-based PRC intervention studies have demonstrated the PRC role’s feasibility and acceptability, reporting significantly higher rates of SU treatment initiation and engagement among patients who received peer-delivered supports compared to those in standard care (30–34).

Integrating PRCs into COPC teams may help shift SU stigma among CHWs and help CHWs patients reduce SU-related barriers to HIV care engagement. In fact, the idea of training peers to provide SU-related supports for PWH emerged organically from our team’s prior work with PWH and lived experience of SU in this setting (18). Despite this, the PRC role represents a workforce innovation for SA, and there are likely to be patient-, provider-, and system-level barriers to the uptake, implementation, and sustainment of this new role. To optimize acceptability, feasibility and contextual appropriateness, we partnered with key stakeholders and used a human-centered design (HCD) process (35, 36) to develop a PRC role that builds off successful US models. This was informed by Designing for Dissemination and Sustainability, an implementation science concept that recommends stakeholder engagement in the design process as a strategy for ensuring that innovations are developed that are aligned with stakeholder preferences and priorities and responsive to potential implementation barriers (37, 38). The aim of this paper is to describe a multi-level stakeholder-driven approach to co-designing a PRC role for integration into COPC teams in Cape Town, SA.

## Methods

### Setting

This study was conducted in low-income communities within the Eastern, Khayelitsha, and Klipfontein subdistricts within Cape Town. These subdistricts are characterized by high rates of poverty, HIV, crime, and SU. Free HIV testing and treatment is available at community health clinics, which oversee COPC teams and other HIV services provided by CHWs. The Western Cape Department of Health (WCDoH) contracts non-governmental organizations (NGOs) to employ CHWs and operate COPC teams (5). Although SU treatment options are limited, there are free or low-cost outpatient programs available in these districts (39).

### Design

**Figure 1:**
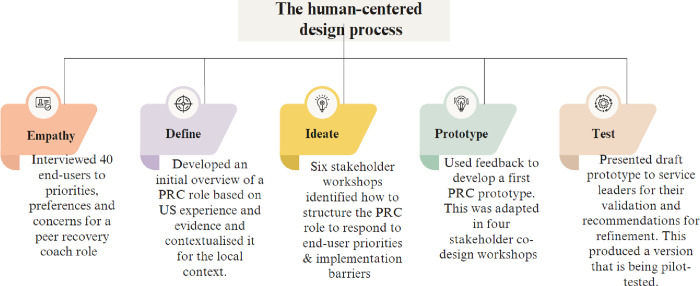
The process of designing a peer recovery coach role for community-oriented primary care teams in South Africa

We chose HCD as our design method as it facilitates the rapid implementation of health system and workforce innovations (36, 40). HCD seeks to understand the implementation context and stakeholders’ design priorities and concerns (41), actively involving stakeholders in the design process (42). Our approach involved five steps (43) shown in Fig. 1: (i) *empathizing* with stakeholders by understanding their priorities and concerns about the proposed role; (ii) *defining* the PRC role and identifying information gaps; (iii) *ideation* of the PRC role and function; (iv) co-designing a PRC *prototype*; and (v) *testing* this prototype via stakeholder consultation, in preparation for pilot implementation of the PRC prototype. As this was an iterative process, the participants and procedures involved in each step are described in the order in which they occurred.

### Participants and procedures

#### Step 1: Exploring stakeholders’ priorities for PRCs

Procedures for Step 1 are described in Magidson, Rose et al. (17). From February to June 2021, 40 semi-structured interviews were conducted (*n* = 15 PWH; *n* = 25 HCWs; Table 1). The WCDoH assisted in identifying HCWs who were at least 18 years old and either provided or managed community-based HIV services, provided clinic-based services but interacted with community-based teams, or provided SU treatment. HCWs identified PWH who were ≥ 18 years old and self-reported difficulties with HIV care engagement and SU.

Trained research assistants (RAs) obtained written informed consent before using a semi-structured interview guide to enquire about SU support needs, acceptability of using peers to address these needs, and potential roles for peers in HIV care teams. Interviews were conducted in isiXhosa or English, lasted approximately 45 minutes, and were audio-recorded, translated into English (if required), and transcribed verbatim.

Guided by thematic analysis (44), we used a hybrid deductive-inductive approach to code the transcripts and develop themes (45). The interview guide was used to develop an initial codebook deductively, with inductive codes added through the open coding of several transcripts. The remaining transcripts were coded by two independent coders, who met weekly to review codes and resolve discrepancies.

#### Step 2: Outlining the PRC role

In February 2022, a US-based design team was formed, comprising (i) a certified PRC Supervisor with experience working as a PRC, and (ii) two US-based RAs with experience working on US-based studies employing PRCs and studies based in SA. First, this team met with the study’s US-based project manager to learn more about the study context and brainstorm how typical functions of the PRC role in the US could inform a SA-based PRC role.

Next, drawing from their experience, best available evidence, and US-based PRC training materials, this team developed an overview of US-based PRC requirements and functions. In the State of Maryland, where the US team is based, PRC are defined as trained and often state-certified individuals with lived experience with substance use and recovery. In this State, individuals in recovery from substance use for two or more years are eligible to apply for PRC certification which requires completion of specialized training in Advocacy, Ethical Responsibility, Mentoring/Education, and Recovery/Wellness, and supervised practice hours. Typical functions of this role in the US include provision of psychosocial support, motivation for recovery, sharing of relevant lived experiences, and service navigation.

In March 2022, this overview was presented to the study’s operations team, comprising three SA-based RAs (with experience working as a peer or non-clinician interventionist on SA-based studies), the SA- and US-based project managers, and a US-based clinical psychology doctoral trainee. Their feedback was used to adapt the overview. Between March and April 2022, the design and operations teams met regularly to tailor the overview to the local context and address stakeholder concerns and priorities identified during *Step 1*. The design team also recorded a brief video where the US PRC Supervisor explained the peer role. This video was shown during *Step 3* workshops.

Meanwhile, the combined US-SA operations team co-developed objectives and HCD questions for these workshops through iterative rounds of feedback. These HCD questions took the form of “how might we” questions and broadly focused on addressing gaps in the team’s understanding of how to define the PRC role within the context of COPC teams, how to structure PRC activities, and criteria to consider when recruiting PRCs.

#### Step 3: Ideation workshops with stakeholders

Between April and June 2022, we conducted six ideation workshops with *n* = 12 patients (across three workshops) and *n* = 12 HCWs (across three workshops); see Table 1 for demographics. Recruitment and eligibility procedures followed those of *Step 1*.

SA-based RAs obtained informed consent and collected participants’ demographic information before the workshops. The workshops began with an overview of the PRC role and the PRC video developed in *Step 2*. Next, RAs used a semi-structured workshop guide that included the HCD questions to elicit feedback on the proposed role. Workshops were conducted in community centers or via online platforms in English, Afrikaans, or isiXhosa (the local languages) and lasted up to an hour. All workshops were audio-recorded, with recordings supplemented by RA notes.

We used a rapid form of qualitative data analysis recommended when engaging in iterative co-design (46). After the workshops, staff reviewed their notes and augmented these with workshop observations. Notes were checked against recordings and supplemented with quotes from participants. The operations team met to rapidly code these augmented notes for key themes and recommendations. To aid interpretation, feedback from all the workshops was distilled into a matrix organized by theme and type of stakeholder.

In May 2022, the project operations team met with a US-licensed clinical psychologist with experience supervising US-based PRCs. During a whole-day in-person meeting, this team collaborated to use this matrix to adapt the PRC overview and develop a prototype for the PRC role in preparation for *Step 4*.

#### Step 4: Prototype co-design workshops

In June 2022, we invited participants from Step 3 to participate in prototype co-design workshops (*Step 4*). We conducted two workshops with HCWs (*n* = 7) and a further two workshops with patients (*n* = 9). The workshops followed *Step 3* procedures. After presenting the PRC prototype, RAs used a semi-structured guide to elicit general feedback on the prototype, adaptations required to enhance acceptability and feasibility, and strategies for addressing contextual barriers. Workshops were conducted in community centers or via online platforms in English, Afrikaans, or isiXhosa, lasting between 45–60 minutes. All workshops were audio-recorded, with recordings supplemented by RA notes. The operations team met to review this feedback, using the approach outlined in *Step 3* to rapidly adapt the PRC prototype (producing version 2) in preparation for *Step 5*.

#### Step 5: Stakeholder consultation to test the prototype

Next, the operations team met with stakeholders responsible for commissioning and implementing COPC teams and clinic-based HIV and SU services. In these consultation meetings, we presented prototype version 2 and requested these leaders to provide feedback on the prototype including suggestions for strategies to support embedding the role within COPC teams. RAs took notes to summarize feedback. The team met to review these notes, using the approach outlined in *Step 3* to further adapt the PRC prototype, producing version 3.

## Results

### Acceptability of PRC and potential roles for the PRC within COPCs (Step 1)

Findings have been published (17) and are only summarized here. Stakeholders described high levels of SU stigma among HCWs, and stigma’s impact on HIV care engagement for people with SU. All stakeholders thought PRCs could offer a unique contribution to COPC by supporting patients with SU recovery and health care navigation. HCWs commented that PRCs could be “*role models*” for patients, showing them that “*SU recovery is possible*.” Patients said they would be more comfortable talking to PRCs about SU compared to HCWs from whom they anticipated stigma. As one patient commented:

I prefer someone who has had the same, has gone through what I’ve also gone through…someone that I can talk to about anything and everything.

Stakeholders also thought that PRCs could help overcome stigma and other HIV care engagement barriers for patients with SU. They reflected that through social contact with HCWs and sharing their lived experience, PRCs could shift HCWs’ attitudes toward patients with SU while demonstrating effective ways of engaging with them. Further, they suggested that having PRCs on the COPC team may make HIV services more “*welcoming*” for people with SU.

Patients and HCWs had different priorities for the role. Patients wanted the defining feature of the role to be the sharing of lived experiences of and support for SU recovery. They also prioritized tailoring the format and content of PRC services to meet individual needs, prioritizing confidentiality of PRC-patient interactions. HCW priorities focused on PRC role clarification to avoid potential role overlap with the CHW role. They also prioritized streamlining PRC activities to fit the current workflow of the COPC team. PRC wellness was an additional consideration for PRC role design, with HCWs raising the importance of PRC training and supervision including strategies to manage contextual and work-related risks to their recovery. This feedback informed planning for the PRC role in *Step 2*.

### Insights from Ideation workshops (Step 3)

Five themes were generated from the Ideation workshops: (i) PRC role expectations and functions; (ii) experience and knowledge prerequisites for PRC role acceptance; (iii) structure and content, (iv) location and implementation of PRC sessions; and (v) essential PRC training components.

### PRC role expectations

All stakeholders were supportive of integrating PRCs into COPCs. They thought PRCs would ease the workload of CHWs who were “*already doing too much*” and address an important SU service gap. These stakeholders recommended that the PRC role involve patient education about SU, its effects on HIV, and SU service options; equipping patients with SU behavior change skills, and supporting PWH to navigate barriers to SU and HIV services.

### Prerequisites for the PRC role

Stakeholders described relevant lived experience, personal recovery, and community knowledge as prerequisites for any PRC. There was broad consensus that lived experience of SU and SU recovery was a role prerequisite. While lived experience of HIV was less salient, stakeholders emphasized the importance of ensuring that PRC training included components focused on HIV. One HCW shared:

I don’t think you only need to focus on a person that is HIV positive… I think it is a person who experienced or dealt with substance use, that is a good person to be part of the team. Because then you can speak from your own experience… that person [the peer] who dealt with it, who has the experience, can be taught [about HIV].

While stakeholders agreed that PRCs did not need to have experience with a particular type of substance, they commented on the importance of PRCs being trained to understand the effects of the different substances being used in their communities. As one patient noted “*Alcohol or drugs [is fine]. They must at least have training [on both]*.” HCWs and patients recommended that the PRC have a minimum of one year in SU recovery. They thought this general guideline would ensure that PRCs had sufficient personal experience of recovery to support others. Several HCWs thought PRCs earlier in their recovery journey would be more vulnerable for relapse when encountering open substance use scenes as they would have less established strategies for managing these environmental risks. As one HCW commented,

I think for a year… there’s no timeline that keeps you safe… So you could be in recovery for ten years, then something, and then you’re back. I just wanted to say a year to give them some time to work on themselves.

While there were some dissenting voices, most stakeholders believed that PRCs should not work in their own neighborhood. HCWs were concerned about the welfare of the PRC, describing the potential for the PRC to be stigmatized by their own community if their SU history became known, and for patients to visit their homes for after-hours assistance. Patients’ concerns focused around confidentiality and dual relationships, with many being more comfortable working with a PRC outside of their community. However, all stakeholders agreed that the PRC would need to be very familiar with the community and share its culture. They described this as a prerequisite for PRC safety (and therefore role feasibility) and for acceptance by the community.

### PRC session structure

HCWs and patients recommended structuring the PRC sessions to include an initial “*intensive support*” phase, characterized by weekly contact sessions lasting up to 30 minutes followed by a less intensive “*step-down*” phase with tapering to bimonthly and then monthly sessions. As a HCW commented:

I would say, in the acute phase, you should obviously have more frequent visits, until you see [the patient] is working with you, cooperating, then you will have less visits…stagger it, so that at least you have a system of, “now you’re in the acute phase, but now you’re going to a less acute phase where you now see less of me and can perform and function on your own.

Stakeholders’ recommendations for the duration of PRC contact ranged from one to six months, recommending that “*the timeframe should be tailored [to the person]*.” After discussion, they agreed it would be feasible to provide PRC sessions tapering in frequency for up to three months.

Stakeholders also discussed session delivery, prioritizing face-to-face contact. While some patients recommended supplementing this with digital or telehealth contact, HCWs were concerned that this would over-burden the PRC as they would be “*on call like 24/7*.” Stakeholders also raised concerns about confidentiality when using digital messaging services, commenting that others might view these messages as mobile phones were often shared within households.

### Session location

Stakeholders provided suggestions for the location of PRC sessions including patients’ homes, community spaces such as libraries, and clinics. Some patients worried that the PRC may be uncomfortable conducting sessions in their homes, suggesting that “*the first few sessions should be at the clinic so that I can tell them about my home situation and they can decide if they want to still come to my house*.” Others raised stigma concerns, stating that PRC home visits (when separate from routine CHW visits), would inform neighbors about their SU. As a result, stakeholders agreed that the PRC should offer patients a choice of session location and that the physical and psychological safety of both the PRC and patient should be considered when selecting the session location.

### PRC training

Stakeholders highlighted knowledge and skills-based competencies to target in PRC training. Stakeholders agreed that training should include topics on HIV and SU, including the effects of various substances, the impact of SU on HIV, SU treatment options and how to support patients to navigate barriers to SU behavior change and services. In addition, stakeholders training on therapeutic competencies related to confidentiality and professional ethics, non-judgmental communication, and therapeutic relationships. Patients emphasized the therapeutic relationship, saying that PRCs “*must bring you on a level that you feel comfortable”* and *“must be trained to build relations*.” HCWs emphasized training on professional work practice, especially for peers new to the healthcare workforce. As one HCW commented:

…the ethics and the professionalism that needs to be brought into training so that people know yes, you’re using substances, but it is not for me to go and tell the whole world that you are in the program.

This feedback shaped the initial PRC role prototype, described in Table 2.

Feedback from prototype co-design workshops (Step 4)

Stakeholders largely endorsed the proposed prerequisites for the PRC role, role expectations, training components, and session structure. Stakeholders recommended augmenting this prototype to include strategies for managing contextual challenges like safety, a description of the PRC’s working conditions, and more detail about PRC session content. Table 2 presents changes to the prototype based on this feedback.

### Feedback from consultation meetings (Step 5)

Stakeholders responsible for commissioning and implementing HIV and SU services endorsed the revised prototype but noted that it did not address the context of the COPC team. HIV experts recommended expanding PRC role expectations to include participation in the broader COPC team and augmenting the proposed training components to include information on COPCs. They also recommended developing PRC integration training for the COPC team to orient them to the PRC role. Further, SU experts recommended augmenting both the PRC role expectations and training schedule to include supervision, peer mentoring and self-care components. They described these components as necessary for helping PRCs navigate potential barriers to their integration within COPC teams, complex community dynamics, and role boundaries while supporting their professional development and safety. Table 2 outlines modifications based on this feedback.

## Discussion

This paper contributes to a small but expanding literature on the value of HCD approaches when designing interventions for dissemination and sustainment (36, 40, 47) by demonstrating the benefits of a stakeholder-engaged, HCD approach to the design of workforce innovations that address the SU service gap in resource-constrained settings. Overall, the multiple stakeholder groups we engaged in the design process thought it was acceptable and feasible to embed PRCs into COPC teams, with the caveat that the PRC role should remain distinct from existing roles within the COPC team. These stakeholders thought the PRC should focus on providing SU recovery and care navigation supports for PWH— services that CHWs do not have the time, training, or capabilities to provide as reported by previous studies (14). Despite this consensus, patients and HCW stakeholders differed in their reasons for wanting the PRC role to remain distinct from that of CHWs within COPC teams.

More specifically, patient stakeholders prioritized the relational components of the PRC role, desiring a better care experience than the one they currently received from CHWs. Like earlier studies (25, 27), patients described their HIV care experience as characterized by SU stigma, poor care quality, and unsupportive provider relationships. These stakeholders hoped that PRCs could offer an alternative care experience by sharing their lived experience of SU recovery to build a strong patient-PRC relationship, and by providing person-centered and confidential support for SU recovery and health care navigation tailored to their personal treatment goals. They also wanted flexibility and choice in how sessions were structured and delivered, similar to patient and peer feedback on the essential elements of PRC services from US-based research (34, 48). During the iterative design process, we used this feedback to modify the PRC prototype to better align with patients’ priorities and preferences. Modifications included (i) expanding PRC role expectations and session content to foreground the sharing of lived SU experiences; (ii) integrating the initial PRC visit with routine CHW visits to the patient’s home to help with initial relationship building and to address SU stigma concerns; and (iii) introducing opportunities for shared decision making about the structure and delivery of PRC sessions.

In contrast, HCW stakeholders prioritized greater definition and clarification of the PRC role, including role boundaries, and suggested modifications to better align the PRC’s work practice with that of the COPC team. They were concerned about potential role duplication and that differences in working conditions, expectations, and practice may increase the amount and complexity of their work. These concerns are not surprising given the unpredictability and insecurity of CHW employment contracts (49) and the well-documented high workload of COPC teams (7). CHWs are unlikely to welcome a PRC into their teams if they think this will make their role redundant or increase the complexity of their work. Role ambiguity has been identified as a barrier to embedding PRCs within US-based primary care (50) and non-specialist providers within primary healthcare more generally (51, 52). In response to these concerns, we modified the PRC prototype to minimize any role duplication by clarifying that CHWs would be responsible for HIV treatment support and PRCs would be responsible for SU-related support, currently not provided by CHWs. Further modifications were made to align the PRC’s proposed working conditions and expectations for professional work practice with those of the COPC team, with PRC training and supervision augmented to address these components.

Role clarification was not a salient concern for HIV and SU service leaders, possibly because they were consulted later in the design process. These stakeholders offered unique insights, based on their health systems experience, into the readiness of COPC teams for PRC integration. They emphasized the need for COPC team members to be educated about the potential contributions of the PRC role and the need to invest in preparing COPC teams for PRC integration. These stakeholders raised concerns about the potential impact on PRC wellness if the COPC team climate was not welcoming, suggesting that the PRC may require additional mentoring and support to navigate these implementation barriers and ensure their psychological safety within the COPC team. These recommendations are aligned with the experiences of introducing peer services for SU and mental health in other contexts (53, 54). Based on this feedback, we added the following components to the PRC prototype: (i) COPC team integration training focused on raising awareness of and fostering openness to the PRC role, (ii) COPC preparation activities to create opportunities for the PRC to build relationships with team members through meetings and work shadowing, (iii) additional training components to orient the PRC to the COPC environment and how to work effectively with the COPC team, (iv) clinical supervision and peer mentoring to support PRCs to deliver high-quality support, navigate dynamics within the COPC team and community, and their psychological safety and wellbeing in complex environments. Earlier studies also identified training and relational work with existing staff as key strategies for supporting the implementation of primary healthcare workforce innovations for mental health (55) and supervision and peer mentoring as important strategies for preventing burnout and protecting the wellbeing of non-specialist health providers in SA(23, 56).

While findings highlight the value of including patient, provider, and systems perspectives, engaging multiple stakeholder groups in co-design is complex and fraught with power imbalances. This study included patients who were marginalised by their socio-economic status, HIV and SU, and CHWs with little power or agency in the healthcare system (5). Further, there were power imbalances within our study team, inherent when adapting an intervention from the global North for application in the global South, and where teams include people with lived experience of SU and other stigmatized identities. To limit the impact of these imbalances on stakeholder inputs, we intentionally used mutual capacity-building (which promotes equitable bidirectional learning) as a guiding framework for stakeholder engagement (57, 58). In all team and stakeholder activities, we worked to ensure that all contributions were equally valued, a diversity of perspectives was encouraged, and everyone felt respected. We distributed leadership and decision-making power across teams and groups, encouraging open discussion, and preferencing stakeholder contributions and the local voice, rather than those of the study team, in design decisions. With an iterative design process, we were able to demonstrate how stakeholder feedback led to prototype modifications, building trust in the design process and enhancing the quality of their contributions. As this approach enriched our design process, we recommend mutual capacity building as a framework for supporting stakeholder engagement in the co-design of SU interventions.

Despite this strength, there are limitations to consider. A relatively small number of patients and HCWs participated in the workshops. Additional engagement with a wider range of patients and HCWs may identify further modifications to the PRC role that could broaden its appeal and utility. Second, the PRC role was designed to be embedded in COPC teams working within disadvantaged communities in the Cape Town metropole where there are high levels of unmet SU-related needs and structural barriers to accessing SU treatment (59). Inter-provincial differences in the organization, function, and funding of COPC teams and unmet need for SU treatment (5, 9) may affect the relevance and utility of the PRC role beyond the current context. Third, we did not quantitatively assess acceptability, feasibility, or appropriateness at each step of the co-design process. Future studies should consider embedding quantitative implementation outcome measures into the co-design process so that the potential utility of the HCD process for enhancing acceptability, feasibility, and appropriateness can be systematically evaluated.

## Conclusion

This paper describes a methodological process that may be useful to other teams developing PRC roles for primary healthcare teams. Through engaging stakeholders with diverse perspectives in the design process, we were better able to understand and respond to their priorities and preferences for the PRC role, enhancing role acceptability and contextual fit. Stakeholder engagement helped identify patient-, provider-, and systems-level barriers to role implementation; the iterative design process allowed us to modify the PRC prototype to enhance role feasibility and contextual fit. Stakeholder engagement also served as an implementation strategy, building collective ownership in the role and helping us identify champions to support the pilot implementation of this workforce innovation. A pilot study (NCT05907174) is underway to evaluate the acceptability and feasibility of embedding the PRC role within COPC teams and preliminary effects on HIV care engagement. While findings from this pilot are likely to inform further modifications to the PRC role prototype, we believe investment in this stakeholder-driven approach to PRC role design will enhance the likelihood of future scaling and sustainment of this role.

## Figures and Tables

**Table 1:** Characteristics of stakeholders participating in the co-design process

	Interviews (Step 1)		Workshops (Steps 3 & 4)	
	*HCWs n*=25 n (%)	*Patients n*=15 n (%)	*HCWs n*=12 n (%)	*Patients n*=12 n (%)
Age – M (SD)	41.2 (8.8)	40.1 (8.2)	42.8 (15.4)	37.5 (8.7)
Female	17 (68%)	11 (73%)	9 (75%)	7 (58%)
Race				
Black African	10 (40%)	15 (100%)	6 (50%)	8 (67%)
Coloured ^a^	12 (48%)	0	5 (42%)	4 (33%)
Other ^b^	3 (12%)	0	1 (8%)	0
Highest Education – n (%)				
< Secondary	3 (12%)	8 (53%)	0	9 (75%)
Secondary	7 (28%)	5 (33%)	2 (17%)	3 (25%)
Some Tertiary	8 (32%)	1 (13%)	4 (34%)	0
University Degree	7 (28%)	1 (13%)	6 (50%)	0
Years in Current Role (HCWs) - n (%)				
< 1 year	1 (4%)	-	4 (33%)	-
1 to 3 years	9 (36%)	-	4 (33%)	-
> 3 years	15 (60%)	-	4 (33%)	-
Employment Status *(Patients)* – n (%)				
Unemployed	-	10 (67%)	-	9 (75%)

a Coloured is an official South African racial category referring to people of mixed-race ancestry who have a unique cultural identity

b Other includes participants who identified as White, Indian, or Asian

**Table 2:** A description of the changes made to the PRC prototype at each stage of the co-design process.

Role Description	PRC prototype (Version 1); produced after ideation workshops *(Step 3)*	Revised prototype (Version 2); produced after co-design workshops *(Step 4)*	Revised prototype (Version 3); produced after consultation with health leaders *(Step 5)*
**Role Prerequisites**	• Lived experience of SU • At least one year of SU recovery. • Lived experience of HIV is not essential • Understanding of community dynamics • Share language and culture of community	*No changes*	*No changes*
**Role expectations and core activities**	• Provide education on HIV and SU • Support patients to connect to and navigate SU and health services • Support to enhance motivation and overcome barriers to SU and health service use	*Added*: • Sharing of personal experience to reduce stigma and instill hope for recovery	*Added*: • Participation in supervision and peer mentorship • Participation in self-care activities • Participation in COPC team activities and meetings
**Working conditions**	*Not addressed*	*Added*: • Safety: PRC to work in pairs or be accompanied by a CHW • Working hours and conditions identical to those of COPC members. • No after-hours contact; respond to messages on next working day. • No uniform due to stigma concerns.	*No changes*
**Core elements of PRC training**	• Confidentiality and professional ethics • Content knowledge: - SU, HIV, and how they relate - Local SU services and how these can be accessed. • Basic counselling skills to support behavior change: - Motivational Interviewing - Nonjudgmental communication - Problem Solving - Behavioral Activation	*Added*: • Training on safe sharing of personal experiences of SU and SU recovery	*Added*: • Information and training on COPCs • Self-care skills to support PRC well-being and recovery • Benefits of supervision and peer mentorship/debriefing
**Structure and content of PRC sessions**	• *Structure*: - One-on-one sessions for 12 weeks - Session 1: Describe PRC role, establish confidentiality and preferences for session format and location. - Weeks 1–4: 1 session per week - Weeks 5–8: 1 session every 2 weeks Weeks 9–12: Patient and PRC to decide on frequency • *Duration*: ~30 minutes, with up to an hour scheduled • *Location*: Initial contact (session one) at patient’s home, COPC will introduce PRC, location of other visits to be decided. • *Mode*: Face-to-face delivery with telephonic delivery if required.	*Added*: • *Structure*: - Session 1: Establish preferences for session times. Clarify boundaries of PRC role and afterhours availability. Share personal experience to engage patient. - Weeks 1–4: Help identify and navigate challenges to engaging in SU/HIV care via education, sharing lived experience and teaching skills for behavior change. - Weeks 5–8: Provide support for recovery and enhance motivation for care engagement, using skills described above. - Weeks 9–12: Provide support for care engagement (if required). Help patient transition from the PRC to other recovery supports in the community (e.g. support groups). • *Location*: Not all patients endorsed an initial home visit due to stigma and safety concerns. To enhance acceptability, the initial PRC session will not occur in a separate home visit. It will be delivered during CHWs' routine visits to the patienťs household.	*No changes*
**Strategies to facilitate PRC integration into COPC teams**	*Not addressed*	*Not addressed*	*Added*: • COPC training prior to integration: - Information on SU, HIV and stigma - Information on PRC role and how it can support the COPC - PRC video - Strategies for supporting COPC wellness - Opportunities to discuss and resolve any concerns • Formal introduction of PRC to COPC at a team meeting • PRC to accompany CHWs on home visits for 8–10 weeks to ensure familiarity with COPC activities and processes, patients, and the community and to build team relationships. • PRC supervision and mentoring
